# Leptospirosis in Hawaii, USA, 1999–2008

**DOI:** 10.3201/eid1702.101109

**Published:** 2011-02

**Authors:** Alan R. Katz, Arlene E. Buchholz, Kialani Hinson, Sarah Y. Park, Paul V. Effler

**Affiliations:** Author affiliations: University of Hawaii, Honolulu, Hawaii, USA (A.R. Katz, K. Hinson);; Hawaii State Department of Health, Honolulu (A.E. Buchholz, S.Y. Park, P.V. Effler)

**Keywords:** leptospirosis, zoonoses, Hawaii, epidemiology, surveillance, bacteria, research

## Abstract

Although infrequently diagnosed in the United States, leptospirosis is a notable reemerging infectious disease throughout developing countries. Until 1995, when the disease was eliminated from the US list of nationally notifiable diseases, Hawaii led the nation in reported annual incidence rates. Leptospirosis remains a notifiable disease in Hawaii**.** To ascertain the status of leptospirosis in Hawaii since the most recent US report in 2002, we reviewed 1999–2008 data obtained from case investigation reports by the Hawaii State Department of Health. Of the 345 case reports related to in-state exposures, 198 (57%) were laboratory confirmed. Our findings indicate a change in seasonal disease occurrence from summer to winter and in the infective serogroup from Icterohemorrhagiae to Australis. Also, during the past 20 years, recreational exposures have plateaued, while occupational exposures have increased. Ongoing surveillance is needed to clarify and track the dynamic epidemiology of this widespread zoonosis**.**

Leptospirosis is considered the most globally widespread zoonotic illness; it has been classified as an emerging or reemerging infectious disease by the World Health Organization (*1*) and the US Centers for Disease Control and Prevention (CDC) (*2*). Most frequently recognized as a disease of the developing world (*3*), leptospirosis was removed from the US list of nationally reportable infectious diseases in 1995 (*4*). Before the disease’s removal from national surveillance, Hawaii consistently led the nation in reported annual incidence rates (*5*). The state of Hawaii continues to include leptospirosis as a notifiable illness. The last published US population-based surveillance report was from Hawaii and covered data obtained during 1974–1998 (*5*). This study serves as an update for leptospirosis in Hawaii during 1999–2008.

## Methods

We reviewed leptospirosis case investigation reports by Hawaii Department of Health (HDOH) investigators submitted during 1999–2008. These reports were (and still are) generated for all reported leptospirosis cases in the state. A standardized case investigation form was used, which includes demographic, epidemiologic, clinical, and laboratory information obtained from patient interviews, medical record reviews, and laboratory reports. Research for this study was approved by the HDOH Institutional Review Board.

For exposure source to be assessed, incubation periods estimated, and exposures classified, patients were asked about high-risk activities that occurred during the 21 days before symptom onset. These included exposure to animals, mud, or potentially contaminated freshwater sources involving occupational activities (e.g., farming, ranching), recreational activities (e.g., freshwater swimming, hiking), or habitational activities (around the home; e.g., gardening, trapping rats). If exposure was continuous or if persons had been exposed multiple times, the incubation period was considered indeterminate. Ascertainment of exposure classification involved placing cases into 3 mutually exclusive exposure categories: occupational, recreational, or habitational. If exposure activities involved >1 category, the exposure classification was considered indeterminate. Outbreaks were defined as >2 epidemiologically linked cases.

A patient with a confirmed case had a clinically compatible illness plus a >4-fold increase in microscopic agglutination test (MAT) titer between acute- and convalescent-phase serum specimens or isolation of *Leptospira* spp. from a clinical specimen (*6**,**7*). All other cases were classified as either probable (clinically compatible illness with MAT titer >200 in >1 serum specimens without a 4-fold increase in titer [*8*]) or suspected (clinically compatible illness with less supportive laboratory evidence of infection [e.g., MAT titer <200, positive macroscopic slide agglutination test result, reactive immunoglobulin (Ig) M ELISA, or positive indirect hemagglutination assay results]). Only laboratory-confirmed cases in patients whose disease was contracted through exposure within the state of Hawaii were included in this analysis. MATs were conducted by CDC from January 1999 through November 2004, and by HDOH from December 2004 through December 2008.

All isolates were sent to CDC for definitive serogroup identification. To determine the presumptive infecting serogroup for serologically confirmed cases, MAT titers were examined. The highest and most recent titer was presumed to be the infecting serogroup. If >1 serogroup had the same high titer, the identification was labeled indeterminate.

To calculate mean annual incidence rates (overall and by age, sex, and Hawaii island on which patient was exposed), the numerator was the number of cases for the specified groups over the 10-year observation period divided by 10. The denominator was the overall or relevant group-specific population estimate from the 2000 US Census (*9*). Data from our earlier 25-year study period, 1974–1998, were used for trend analyses (*5*).

We calculated frequencies, tests for trends, and tests for difference using Epi Info version 3.3.2 (CDC, Atlanta, GA, USA); p values <0.05 were considered significant. All statistical tests were 2-tailed.

## Results

HDOH received 356 leptospirosis case reports; 345 were related to exposures within the state of Hawaii. The 11 cases from exposures occurring out of state included 2 from Guam; 2 from Thailand; and 1 each from Panama, the Federated States of Micronesia, Borneo, Okinawa, Malaysia, Singapore, and Texas. Of the case reports related to in-state exposures, 198 (57%) were laboratory confirmed, 116 (34%) were probable, and 31 (9%) were suspected.

**Figure 1:**
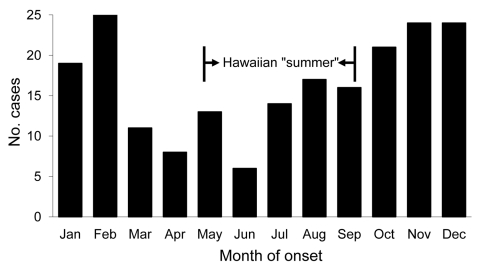
Month of onset for 198 laboratory-confirmed leptospirosis cases, Hawaii, USA, 1999–2008.

The number of confirmed cases reported per year ranged from 11 to 27 (median 20), and the estimated mean annual incidence rate was 1.63 per 100,000 population. Mean monthly reported cases were highest from October through February (Figure 1). The observed seasonal disease occurrence for the recent 10-year study period was significantly different from that of the previously reported 25-year study period; summer cases predominated in the latter (p<0.01) (*5*).

**Figure 2:**
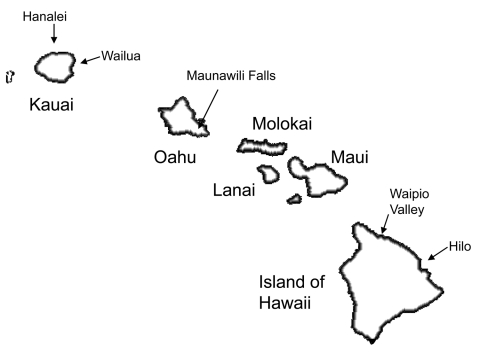
Exposure locations associated with the greatest number of leptospirosis cases, Hawaii, USA, 1999–2008.

Case-patients were predominately male (91%), and ages ranged from 3 to 76 years (median 38 years). The highest age-specific rate was among persons 20–29 years of age, and the lowest was among children 0–9 years of age. Most cases and the highest incidence rates were related to exposures on the islands of Kauai and Hawaii (Table 1). In addition, cases were most consistently reported from the northeast, windward sides of the islands: Hanalei (n = 8) and Wailua (n = 12) on Kauai, Waipio Valley (n = 12) and Hilo (n = 17) on Hawaii, and Maunawili Falls (n = 13) on Oahu (Figure 2).

**Table 1:** Sex, age, and island of exposure for 198 case-patients with laboratory-confirmed leptospirosis, Hawaii, USA, 1999–2008

Variable	No. (%) case-patients	Estimated mean annual incidence rate*
Sex		
M	181 (91)	2.97
F	17 (9)	0.28
Age group, y		
0–9	1 (1)	0.06
10–19	24 (12)	1.46
20–29	48 (24)	2.87
30–39	31 (16)	1.69
40–49	38 (19)	2.05
50–59	36 (18)	2.55
60–69	15 (8)	1.68
70–79	5 (3)	0.64
Island		
Hawaii	98 (49)	6.59
Kauai	47 (24)	8.06
Oahu	44 (23)	0.50
Maui	4 (2)	0.34
Molokai	1 (1)	1.38
Unknown	4 (2)	

*No. cases/100,000 population. Rate = no. case-patients observed over 10 years for the specified category divided by 10 divided by specified subgroup population estimate from 2000 US Census data (*9*).

**Figure 3:**
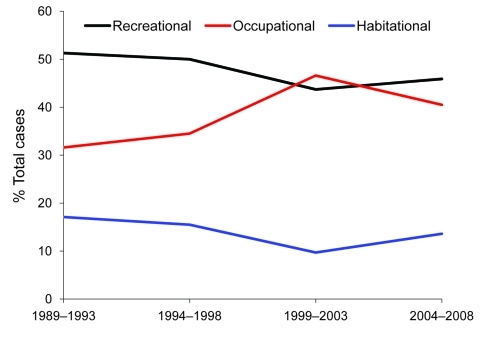
Trends in exposure classification for laboratory-confirmed leptospirosis cases, Hawaii, USA, 1989–2008**.**

We were able to determine exposure classifications for 177 (89%) of the 198 confirmed cases. Recreational exposures accounted for 79 (45%) and were mostly related to freshwater swimming, hiking, and camping. Occupational exposures accounted for 78 (44%), mostly relating to farming, specifically, taro farming. Exposures around the home accounted for 20 (11%), most commonly, gardening. After categorizing cases into 5-year intervals and comparing the results with reports from 1989 through 1998 (*5*), we found that recreational exposures remained relatively stable over the past 20 years (1989–2008), while occupational exposures actually increased, but the difference was not significant (p = 0.08) (Figure 3). After stratification by island, a significant increase in occupational exposures was shown for the island of Hawaii (p = 0.04). No other trends for exposure classification were significant.

Most cases occurred sporadically. One outbreak (>2 epidemiologically linked cases), which involved 2 landscapers, occurred on Kauai in 1999; both cases were laboratory confirmed. Another outbreak (2 epidemiologically linked cases: 1 confirmed, 1 probable) was associated with flooding of the University of Hawaii campus on October 31, 2004, when heavy rains caused an adjacent stream to overflow its banks (*10*).

For case-patients with known exposure dates, the median incubation period was 9 days (range 1–21 days). The median duration of illness was 14 days (range 3–90 days). A total of 118 (73%) of 161 case**-**patients, for whom treatment information was available, were hospitalized.

The most frequent signs and symptoms among patients who sought treatment were fever, myalgias, headache, nausea, and vomiting. Abnormal urinalysis results were common; specimens from 78 (73%) of 107 and 71 (68%) of 105 case-patients showed hematuria and proteinuria, respectively. Results of liver function tests were frequently abnormal as well; laboratory results for 109 (74%) of 147 case-patients showed elevated alanine aminotransferase levels (>40 U/L), and 85 (63%) of 134 showed elevated total bilirubin levels (>1 mg/dL). The most common hematologic anomaly was thrombocytopenia (<140 × 10^9^/L), which was observed for 97 (66%) of 146 case-patients (Table 2). Initial clinical impression was recorded for 151 (76%) of 198 patients. The most common initial diagnosis was leptospirosis for 114 (75%) of 151 patients.

**Table 2:** Clinical findings for 198 case-patients with laboratory-confirmed leptospirosis, Hawaii, USA, 1999–2008

Sign, symptoms, and laboratory result	No. (%) patients affected	No. patients with data available
Sign or symptom		
Fever	187 (98)	191
Myalgia	162 (88)	185
Headache	156 (87)	179
Nausea	117 (68)	173
Vomiting	101 (59)	172
Arthralgia	73 (46)	157
Diarrhea	79 (46)	171
Backache	50 (34)	146
Jaundice	53 (33)	163
Oliguria or anuria	32 (21)	152
Conjunctival suffusion	30 (19)	156
Nuchal rigidity	23 (14)	159
Pneumonia	13 (8)	154
Hepatosplenomegaly	8 (6)	140
Laboratory results		
Renal		
Hematuria	78 (73)	107
Proteinuria	71 (68)	105
Elevated creatinine (>1.5 mg/dL)	60 (51)	118
Elevated blood urea nitrogen (>20 mg/dL)	68 (50)	136
Hepatic		
Elevated alanine aminotransferase (>40 U/L)	109 (74)	147
Elevated total bilirubin (>1 mg/dL)	85 (63)	134
Hematologic		
Thrombocytopenia (<140 × 10^9^/L)	97 (66)	146
Elevated leukocyte count (>10 × 10^9^ cells/L)	74 (48)	155
Decreased hematocrit (<34%)	56 (38)	146

During the 10-year reporting period, 1 death occurred among 198 patients with confirmed cases (case-fatality rate 0.5%). A 23-year-old man who attended college on the mainland had been exposed through recreational activities while at home in Hawaii during winter break 2003. Symptoms developed after he returned to school, and he died in January 2004.

Of the 198 patients with confirmed infection, 152 (77%) received a diagnosis on the basis of serologic testing with the MAT, 18 (9%) cases were confirmed with culture isolates, and 28 (14%) were confirmed by MAT and isolates. Forty-three isolates obtained during 2000–2008 were characterized at CDC by molecular and serologic techniques. Isolates were grouped into 4 clades based on MAT results and pulsed-field gel electrophoresis: 19 (44%) unknown serovar (serogroup Australis), 17 (40%) serovar Icterohemorrhagiae (serogroup Icterohemorrhagiae), 4 (9%) serovar Ballum (serogroup Ballum), and 3 (7%) of unknown serovar (serogroup Bataviae). Cross-agglutination absorption assay identified the unknown serovar from serogroup Australis as a new serovar closely related to Lora (*11*).

The most common infecting serogroups (identified definitively by isolate or presumptively by MAT) were Australis (n = 50) and Icterohemorrhagiae (n = 51). Analysis for linear trend, after cases were categorized into 5-year intervals and compared with confirmed cases reported during 1974–1998 (*5*), showed a significant increase in infections attributed to serogroup Australis and a decrease in infections caused by serogroup Icterohemorrhagiae (p<0.0001 for each).

## Discussion

The most recent 10-year reporting period has demonstrated a statistically significant shift in the seasonal occurrence of leptospirosis from the drier summer months (*5*) to the wetter winter months. Climatologists have characterized the Hawaiian archipelago as having only 2 seasons: summer (May through September) and winter (October through April). Rainfall and widespread rainstorms are most common during the winter months (*12*).

During the earlier reporting period, 1974–1998, recreationally associated exposures predominated and increased over time; therefore, the summer predominance was attributed to the greater likelihood of recreational exposure in the summer. During 1989–2008, the frequency of recreational exposures plateaued while frequency of occupational exposures seemed to increase. This observed change in exposure history might allow seasonal climatic effect to have a greater influence on the epidemiology of the disease. In addition, taro farming, a recognized high-risk occupation (*5*), which had been on the decline, has experienced a resurgence relating to renewed interest in the cultural importance to native Hawaiians and an awareness of taro’s nutritional value (*13*). In 2000, Hawaii produced 7 million pounds of taro, the largest crop yield since 1977 (*14*).

The island distribution of leptospirosis cases remains virtually unchanged since our earlier report (*5*). Kauai, the island with the highest annual rainfall and second most rural island, had and continues to have the highest incidence rate, followed by Hawaii, the most rural island. As in our earlier report, cases were most consistently reported from the wetter, windward, northeast sides of each island. Notably, climatic changes have been documented for the Hawaiian archipelago with significant trends in increasing temperatures (*15*), decreasing rainfall (*16*), and increasing rain intensity (*17*) over the past 30 years. The effects of climate change on ecosystems are complex, but the potential for influencing infectious disease patterns has been well described (*18**,**19*). Temperature and climate changes may affect the host animal’s environment, making transmission to humans more likely. Increase in rain intensity with resultant flooding is a well-recognized climatic risk factor for transmission of *Leptospira* spp (*20*). Flooding was responsible for 1 of the 2 outbreaks during the study period (*10*).

The predominance of men among case-patients is well recognized (*21**–**24*) and is virtually unchanged from our earlier report (5). This predominance has been explained by the tendency of more men to participate in high-risk outdoor exposure activities. The low reported age-specific case rates in children <10 years of age and highest rates among adults 20–50 years of age are also consistently reported (*22**–**24*) and similar to our earlier findings (5).

Our findings corroborate other large case series that show that the most common clinical manifestation of leptosporisis are nonspecific signs or symptoms, such as fever, headache, and mylagias (*5**,**22**,**25**–**27*). The case-fatality rate (0.5%) is lower than that reported from Brazil (*25*), Barbados (*28*), Guadeloupe (*22*), and the Andaman Islands (*26*), but similar to the rates found in our earlier study (*5*) and in a recent case series from France (*27*). The low case-fatality rate in this series may be explained by early recognition and initiation of supportive therapy and antimicrobial drugs. Other case series may be biased toward recognition and inclusion of only the most severely ill, hospitalized patients, which leads to higher case-fatality rates. A recent population-based case-control study from Brazil (*29*) showed that pulmonary involvement was the strongest independent predictive factor for death caused by severe leptospirosis. Pulmonary findings were infrequent among case-patients in this study, the earlier Hawaii series (*5*), and the France series (*27*).

The changing temporal trend in the infecting serogroup first identified in our earlier study has continued; most of the current leptospirosis isolates are in the Australis serogroup. This documented trend over the past 35 years from the previously predominant serogroup Icterohemorrhagiae to the now predominant Australis may reflect the influence of different host animals, the effects of climatic and land use changes, or both. Serogroup Icterohemorrhagiae has been associated with rats (*Rattus norwegicus* and *R. rattus*), and Australis has been associated with swine, including feral swine or wild boars (*Sus scrofa*) (*30**,**31*). Recent reports from Germany have shown high seroprevalence of Australis serogroup (serovar Bratislava) in urban feral swine (*32*) and documented increased size in the feral swine population and habitat changes leading to epidemiologic linkages between leptospirosis occurrence and feral swine exposure (*33*). Hawaii has also experienced an increase in the feral swine population, with a concordant sharp increase in the number of feral swine encroaching on urban residential areas (*34**,**35*). Researchers at the University of Hawaii are currently investigating the possible influence of feral swine exposure on human disease in Hawaii by undertaking a leptospirosis seroprevalence study of feral swine.

Annual reported leptospirosis incidence rates in the United States ranged from 0.02 to 0.05 per 100,000 population from 1974 through 1994, the last year leptospirosis was included in the list of nationally notifiable diseases (*5*). If we include probable and suspected cases, as was done nationally, our mean estimated annual incidence rate during this 10-year study period would increase from 1.63 to 2.85 per 100,000 population, ≈100× greater than that reported nationally. Compared with other locales for which annual leptospirosis incidence rates are available, Hawaii would be considered in the moderate range category (1–10/100,000 population) (*36*). Countries in this range include Cuba (2.47/100,000 population) and Costa Rica (6.72/100,000 population) (*3*). Countries categorized as having high rates (>10/100,000 population) include Barbados (10.03/100,000 population), Trinidad and Tobago (12.04/100,000 population), and Seychelles (43.21/100,000 population) (*3*). Additional countries or regions considered to have high rates (for which data are not available) are Vietnam and French Polynesia (*36*). Although leptospirosis is a notifiable disease in Hawaii, case reporting is based on passive surveillance and likely underestimates true disease occurrence. During a 1-year period in 1988 and 1989, an active surveillance study was conducted on the islands of Hawaii and Kauai, which resulted in a 5-fold increase in case identification (*37*). A recent retrospective analysis of serum obtained from febrile patients during a dengue fever outbreak in Hawaii, 2001–2002, also identified a substantial number of leptospirosis cases that otherwise would have gone undiagnosed (*38*).

## Conclusions

Future field studies using geographic information system technology to link climatic and environmental phenomena, such as rainfall occurrence and environmental isolates with human and animal infection, could offer valuable insights. Given the potential effects of climate and land use changes, public health officials must remain alert to the occurrence and changing epidemiology of emerging and reemerging infectious diseases. Without national surveillance, the occurrence of leptospirosis outside of Hawaii or other regions that have leptospirosis surveillance may go largely unrecognized, and thus, unmonitored. Ongoing surveillance activities, such as ecologic, animal, and laboratory studies are necessary to clarify and track the dynamic epidemiology of this widespread, reemerging zoonotic illness.
